# Molecular characterization of a cDNA encoding Cu/Zn superoxide dismutase from *Deschampsia antarctica *and its expression regulated by cold and UV stresses

**DOI:** 10.1186/1756-0500-2-198

**Published:** 2009-09-28

**Authors:** Jaime R Sánchez-Venegas, Jorge Dinamarca, Ana Gutiérrez Moraga, Manuel Gidekel

**Affiliations:** 1 Laboratorio de Biología Molecular Aplicada, Instituto de Agroindustrias, Facultad de Ciencias Agropecuarias y Forestales, Universidad de La Frontera, Casilla 54-D, Temuco, Chile; 2 VentureL@b, Escuela de Negocios, Universidad Adolfo Ibáñez, Av. Diagonal Las Torres 2700, Peñalolén, Santiago, Chile

## Abstract

**Background:**

The Copper/Zinc superoxide dismutase (Cu/ZnSOD) gene, *SOD *gene, was isolated from a *Deschampsia antarctica *Desv. by cDNA library screening. The expression of SOD gene in the leaves of *D. antarctica *was determined by RT-PCR and its differential expression of gene transcripts in conditions of cold and UV radiation stresses was revealed by northern blot.

**Findings:**

The molecular characterization shows that *SOD *cDNA is 709 bp in length, which translates an ORF of 152 amino acids that correspond to a protein of predicted molecular mass of 15 kDa. The assay shows that the expression of *SOD *gene increases when *D. antarctica *is acclimatised to 4°C and exposed to UV radiation. These results indicate that the *SOD *gene of *D. antarctica *is involved in the antioxidative process triggered by oxidative stress induced by the conditions of environmental change in which they live.

**Conclusion:**

The present results allow us to know the characteristics of Cu/ZnSOD gene from *D. antarctica *and understand that its expression is regulated by cold and UV radiation.

## Background

Superoxide dismutase (SOD; EC 1.15.1.1) is a ubiquitous enzyme belonging to a family of metalloenzymes, which catalyze the dismutation of superoxide radicals to hydrogen peroxide and molecular oxygen. The reaction is continued by catalase (CAT) and ascorbate peroxidase (APX), converting the hydrogen peroxide to water and molecular oxygen, thus preventing the formation of hydroxyl radicals, which are highly destructive to the cell [1,2]. The enzyme SOD is considered the first-line defense because it catalyses the first reaction in the system harvesting oxygen-free radicals [3]. Therefore, SOD prevents the oxidation of biological molecules, performed by the same radicals or their derivatives [4].

Three types of SOD have been characterized based on the nature of the metal co-factor present in the catalytic site, these are: SODs with copper/zinc (Cu/ZnSOD) [5], iron (FeSOD) [6] or manganese (MnSOD) [7]. In cell plant, the Cu/ZnSOD is generally found in the chloroplasts, the cytosol, and possibly in the extracellular space. MnSOD is location in the mitochondria and the peroxisome; whereas FeSOD is present within the chloroplasts of some plants [8,9]. The first Cu/ZnSOD genes were cloned from maize and pea plants [10,11]. SOD sequences of other plants were subsequently cloned, such as snuff [12], tomato [13], rice and poplar [14,15]. It has been observed that SOD activity in plants increases in response to a variety of chemical and environmental stimuli (e.g., arsenate stress, exposure to air pollution, high light intensity, low temperature, low lighting, treatment with agents that generate oxygen radicals, etc.) [16-19].

*Deschampsia antarctica *Desv. is a grass specie adapted to the extreme climate of the Maritime Antarctic [20], presenting tolerance to low temperatures usually between -2°C to 6°C in summer and freezing in winter time [21], episodes of high light and increased UV radiation (because of the thinning of the ozone layer) during spring [22,23]. It has been determined that the combination of low temperature, high light and UV radiation leads to increased overproduction of reactive oxygen species such as superoxide anion, hydrogen peroxide and hydroxyl radicals [24,25]. The accumulation of these free radicals in cells produces lipoperoxidation in membranes, ruptured DNA and enzyme inactivation [3]. The defense system of the cell, composed of enzymatic and non-enzymatic antioxidants, minimizes the deleterious effects of the free radicals [26]. Among the most important antioxidants are the enzymes SOD, CAT and APX. Experiments carried out in the laboratory with *D. antarctica *show that there is an increase in the activity of antioxidant enzymes SOD and APX when subjected to high light and acclimatization of these plants at 4°C [27]. Therefore, *D. antarctica *tolerates high levels of oxidative stress, most likely by expressing genes that confer tolerance to adverse environmental conditions. This tolerance to a wide variety of restraining environmental factors has been correlated with increased antioxidant enzyme activity, as well as antioxidant metabolite levels [27,28].

In this report, we describe the molecular characterization of the Cu/ZnSOD gene of *D. antarctica *and analyzed their expression under stress conditions at low temperatures (acclimation at 4°C) and exposure to UV radiation.

## Methods

### Plant material and growing conditions

*Deschampsia antarctica *Desv. (Poaceae) plants were collected in the Coppermine Peninsula on Robert Island, Maritime Antarctic (62°22'S; 59°43'W) and transported to the laboratory. Leaves samples were collected on-site and immediately frozen in liquid nitrogen from plants exposed to ambient light (subjected to UV) and plants that were kept under non UV conditions for 14 days by using polyester filters (DuPont Mylar, USA). The samples were kept at -80°C in the laboratory. Another group of plants from the same area and at the same developmental stage was placed in plastic containers and transported to Chile. These plants were propagated vegetatively in plastic pots using a soil:peat mixture (3:1), fertilized with 0.12 g/l phostrogen (Solaris, Buckinghamshire, UK) once every 2 week. These plants were cultivated under controlled conditions simulating Antarctic conditions. The laboratory conditions were 13°C temperature (non-acclimatized) or 4°C (acclimatized) under a photoperiod of 21/3 h light/dark and 60-70% relative humidity. Cool-white fluorescent tubes F40CW (general Electric, Charlotte, NC, USA) were used as light source.

### Preparation of gene probes

The *SOD *gene present in the cDNA library of *D. antarctica *was used as a probe. The *SOD *gene was obtained by PCR from the pAGEN-1 vector (Agencourt), using the primer: forward pSOD and reverse pSOD, and following the same conditions used in the amplification of RT-PCR. The product obtained from 475 bp was cloned into pGEM-T Easy vector (Promega) according to the supplier's recommendations and forming the construction pGEM-T Easy-SOD. The insert used as a probe was released from the plasmid with the restriction enzymes *Eco*R I and *Xba *I. The α-tubulin gene present in the pGEM-T Easy-α-tubulin construction was used as a probe for loading control. The SOD and α-tubulin probes were labeled with ^α32^P-dCTP using the HexaLabel™ DNA Labeling Kit (Fermentas).

### Screening of the cDNA library

The Laboratory of Applied Molecular Biology at the Universidad de La Frontera, holds a normalized cDNA library of *D. antarctica *constructed from Antarctic plants and cloned into the pAGEN-1 vector (Gidekel *et al*, non published data).

The scrutiny was done according to the procedure described by Sambrook and Russell [29]. Using a partial cDNA probe of the SOD gene derived from a *D. antarctica*'s subtractive library, it was determined that the clone C13 corresponded to a superoxide dismutase gene (*SOD *gene).

### Analysis of the cDNA sequence

The *SOD *gene cloned into pAGEN-1 vector was sequenced using the universal primers T7 and SP6. The sequencing was performed on an ABI 3730XL sequencer (Applied Biosystems) at Macrogen (Korea). The sequence of the *SOD *gene from *D. antarctica *was analysed using the Vector NTI 7 software (Invitrogen) to predict the isoelectric point (p*I*) and the molecular weight. Homology searches were carried out using the BLAST program  on the NCBI web-server. Multiple sequence alignments were constructed with the program DS Gene 1.5 (Accelrys).

### Extraction of total RNA

For each stress condition to which *D. antarctica *was subjected, total RNA was extracted using the reagent Chomczynski (Winkler Ltda) and following the manufacturer's recommendations. Briefly, the technique was established mixing 1 ml reagent Chomczynski (phenol-guanidine thiocyanate) with 0.2-0.3 g of plant tissue pulverized in mortar with the help of liquid nitrogen. The mix were incubated for 5 min at 4°C, insoluble material was removed by centrifugation at 12000 × g for 10 min at 4°C and the aqueous phase was transferred to a new Eppendorf tube. 200 μl of chloroform was added to the aqueous phase and was shaken vigorously for 15 s. After incubating for 3 min at 4°C, it was centrifuged to 12000 × g for 15 min at 4°C (this step was repeated to obtain clean material). The aqueous phase was transferred to a new tube and RNA was precipitated with 500 μl isopropanol incubating samples at 4°C for 10 min. It was centrifuged for 10 min at 12000 × g to 4°C and the supernatant was removed. The pellet was washed with 1 ml of 75% ethanol, centrifuging at 7500 × g for 5 min at 4°C. The RNA pellet was dried at room temperature and dissolved in 50 μl deionized water treated with diethyl pyrocarbonate to 0.1%.

### Extraction of genomic DNA

For use as a control in the process of determining mRNA *D. antarctica *by RT-PCR, genomic DNA was extracted from fresh leaves, using the reagent Chomczynski (Winkler Ltda) and following the supplier's recommendations. This technique was performed mixing 1 ml Chomczynski with 0.2-0.3 g of plant tissue pulverized in mortar with the help of liquid nitrogen. The mixture was centrifuged to 10000 × g for 10 min at room temperature and DNA in the supernatant was transferred to another Eppendorf tube to mix with 200 μl of chloroform. The DNA was recovered by centrifugation, using the same conditions as the first step, and transferring the supernatant to a new tube. The DNA was precipitated with 500 μl of absolute ethanol, mixing by inverting at room temperature for 1-3 min. The pellet of DNA was obtained by centrifuging at 4000 × g at room temperature for 2 min and was washed with 95% ethanol by simple spin. The extracted DNA was dried at room temperature and resuspended in 100 μl of deionized water free of DNAsas.

### RT-PCR analysis

To study SOD expression in response to stress, the RT-PCR method was employed. Briefly, 1 μg of total RNA was used as template and 500 ng of oligo dT primer (Fermentas) were incubated at 70°C for 10 min in a Peltier Thermal Cycler, PTC-200 (MJ Research, USA). The RNA was incubated on ice for 5 min and 4 μl 5× buffer,1 μl (10 U) of RiboLock Ribonuclease Inhibitor (Fermentas), 2 μl of dNTPs (10 μM) and 1 μl of 0.1 M DTT (Fermentas) were added. The mix was incubated at 37°C for 5 min and was immediately kept on ice for 5 min. Subsequently, 400 U of RevertAid™ F minus M-MuLV Reverse Transcriptase (Fermentas) enzyme was added and incubated at 37°C for 60 min; the enzyme was then inactivated by heating at 70°C for 10 min. Finally 0.5 μl of Ribonuclease A (10 mg/ml) (Fermentas) was added and incubated at 37°C for 20 min.

Conventional PCR was carried out on 5 μl samples generated cDNA by adding 1.5 μl of 5 mM MgCl_2_, 5 μl of 10·PCRbuffer, 1 μl of 10 mM dNTP mix, 1 μl of each specific primer (10 pmol), 2.5 μl of Taq DNA polymerase (1 U/μl) (Fermentas), and water to 25 μl. PCR amplification reactions were initially incubated at 95°C for 5 min, followed by 25 cycles at 95°C for 45 s, 58°C for 30 s, and 72°C for 50 s, a final elongation for 5 min at 72°C was used. Reaction products were analyzed by gel electrophoresis. Each RT-PCR experiment was performed at least three different times to ensure that the results were reproducible. As a control for the PCR reaction, 300 ng of genomic DNA of *D. antarctica *and specific primers for the *psbA *gene were used. As a positive control 50 ng of the plasmid DNA from *E. coli *Top 10F'::pGEM-T Easy-SOD was used. The gene-specific primers used for the PCR were forward pSOD 5'-AAGAATTCATGGTGAAGGCTGTAGCTGTGC-3', reverse pSOD 5'-ATATTCTAGACCCTGGAGCCCGATGATCC-3'; these primers amplified a 475-bp product from the *SOD *cDNA.

### Northern blot analysis

Northern blot analysis was performed according to the protocol described by Sambrook and Russell [29]. For this purpose, 20 μg of total RNA from *D. antarctica *leaves under various stress conditions were electrophoresed on denaturing formaldehyde-1% agarose gel. The fractionated RNA was transferred to a nylon membrane Hybond-N^+ ^(Amersham Pharmacia Biotech, Buckingham, England), exposed to UV radiation on both sides to 1200 μjoules ×100 in a Stratalinker 1800 (Stratagene, La Jolla, CA, USA) and hybridized with each cDNA fragment marked with ^α32^P-dCTP. The hybridization was performed at 65°C for 18 h in hybridization solution (500 mM Na_2_HPO_4 _pH 7.5, 7% SDS and 1% BSA). To eliminate the radioactive probe, the membranes were washed twice with a solution of high stringency (100 mM Na_2_HPO_4 _pH 7.5, 0.5% SDS and 1 mM EDTA) and twice with a solution of low stringency (40 mM Na_2_HPO_4 _pH 7.5, 0.5% SDS and 1% EDTA). Each wash was performed for 15 min at 65°C and the membrane was exposed to X-ray film (Kodak, NEN Life, USA) at -70°C for 15 days. The bands were visualized after developing X-ray films.

The membrane was regenerated by treatment with a solution of hot 0.5% SDS and incubated for 1 h at 65°C with constant agitation. Then repeated the process left to cool to room temperature and to remove excess SDS was rinsed with 40 mM Na_2_HPO_4 _pH 7.5. Subsequently the regenerated membrane was hybridized with the probe corresponding to the constitutive gene α-tubulin used as loading control. The hybridization with total RNA present in the regenerated membrane was performed as described above. The densitometric analysis of the autoradiograms was performed using the ImageJ program (NIH).

## Results and Discussion

### Isolation and sequencing of a cytosolic Cu/ZnSOD cDNA

**Figure 1:**
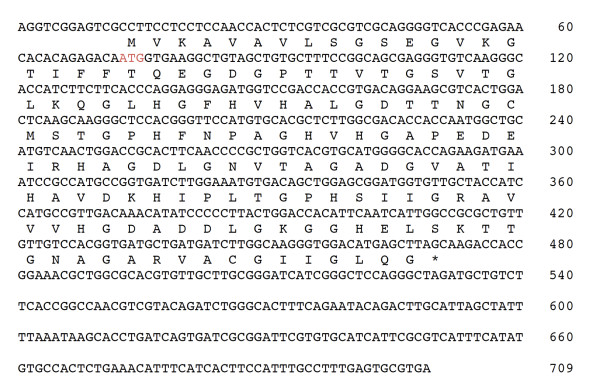
**Nucleotide sequence of the cDNA encoding SOD and amino acid sequence**. The nucleotide sequence showing the ORF of *SOD *gene and above of the nucleotide sequence is shown the deduced amino acid sequence of the putative protein SOD in one-letter code. The start codon of translation is denoted in red and asterisk denotes the stop signal.

After the screening of the cDNA library of *D. antarctica*, the clone C13 was found to contain the superoxide dismutase gene (*SOD *gene). The *SOD *gene cDNA has a length of 709 bp with an open reading frame (ORF) of 456 bp, corresponding to 152 amino acids. This *SOD *gene, encodes a protein with a predicted molecular mass of 15.1 kDa and a p*I *of 6.15. The *SOD *cDNA have the start and finish codons, indicating that the gene is complete (Figure 1). The sequence for the secretion signal peptide was not detected, which leads us to assume that the *SOD *gene probably corresponds to a protein located in the cytosol.

**Figure 2:**
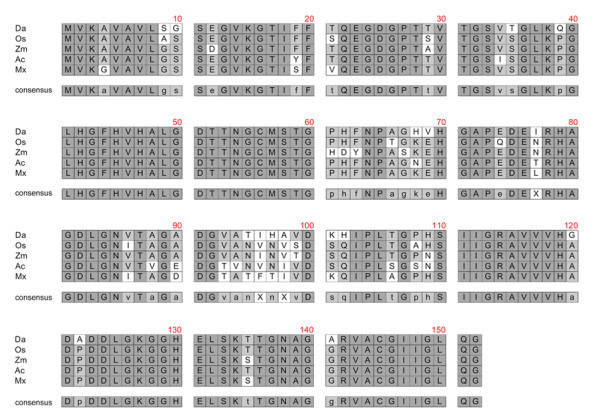
**Multiple alignment of the protein sequence derived from the cDNA sequence *SOD *with other plant SODs from the GenBank databases**. The multiple alignment of the amino acid sequence was done using Accelrys DS Gene 1.5 software. (Da) Putative Cu/ZnSOD of *Deschampsia antarctica*, (Os) Cu/ZnSOD of *Oryza sativa*, (Zm) SOD-Sod4A of *Zea mays*, (Ac) Cu/Zn-SOD-Sod1 of *Ananas comosus*, (Mx) SOD-Sod1 of *Malus xiaojinensis *and the region of consensus.

The deduced amino acid sequence of the SOD product was compared with those of other plant cytosolic SODs available from GenBank. It displayed high homology to a Cu/ZnSOD from *Oryza sativa *[Acc No. D01000], SOD-Sod4A from *Zea mays *[Acc No. X17564], Cu/ZnSOD-Sod1 from *Ananas comosus *[Acc No. AJ250667] and SOD-Sod1 from *Malus xiaojinensis *[Acc No. AY646367] (Figure 2, Table 1). The predicted molecular weight and isoelectric point of the Cu/ZnSOD from *D. antarctica *do not differed significantly in comparison to other plants SODs (Table 1).

**Table 1:** Comparison of amino acid sequence homology and biochemical properties of SODs from different sources with SOD of *D. antarctica*

**SOD plant species**	**Amino acid**		**Biochemical properties**	
	
	**Similarity** **(%)**	**Identity** **(%)**	**Mw** **(kDa)**	**Isoelectric point**
*Deschampsia antarctica*	-	-	15.132	6.15
*Orysa sativa*	90.1	84.9	15.080	5.93
*Zea mays*	88.2	84.2	15.088	5.46
*Malus xiaojinensis*	90.1	85.5	15.105	5.77
*Ananas comosus*	88.2	83.6	15.175	5.30

Mw: Molecular weight

### Differential expression of the Cu/ZnSOD gene under stress conditions

**Figure 3:**
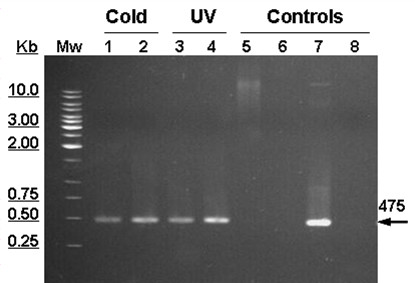
**RT-PCR analysis of Cu/ZnSOD expression in response to cold and UV stresses conditions**. RNA was extracted from leaves of plants subjected to stress conditions. Plants cultivated in laboratory at 13°C (not acclimated) and at 4°C (acclimated) (Lane 1 and 2). Plants growing in the Antarctic without UV radiation and under ambient light (UV radiation) (Lane 3 and 4). Controls: genomic DNA of *D. antarctica *(300 ng) (Lane 5), RNA at 4°C (acclimated) (Lane 6) with primers specific to amplify psbA gene. DNA from *E. coli*::pGEM-T Easy-SOD clone (50 ng) (Lane 7) was used as positive control. Deionised water, negative control (Lane 8). MW: Molecular weight marker (Fermentas). Reverse transcription of 1 μg samples of total RNA was carried out, followed by conventional PCR amplification using gene-specific primers. Reaction products (20 μl) were analyzed by gel electrophoresis.

By means of RT-PCR, the expression level of the *SOD *gene was determined in samples of *D. antarctica *leaves exposed to various stress conditions: plants kept in the laboratory at 13°C (non-acclimated) and 4°C (acclimated); plants obtained in the Antarctic grown under ambient light (with UV) and plants grown under UV filters (without UV). The results obtained by non-quantitative RT-PCR, showed that mRNA of the *SOD *gene of *D. antarctica *is differentially expressed under the stress conditions to which the plant was subjected (Figure 3).

**Figure 4:**
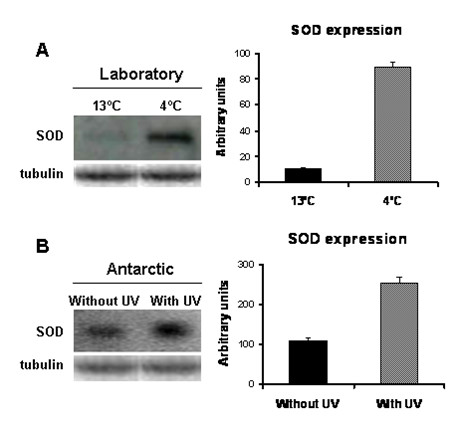
**Northern blot analysis of expression of the Cu/ZnSOD gene in from *D. antarctica***. Changes in the levels of Cu/ZnSOD transcripts in *D. antarctica *plants subjected to cold (A) and UV (B) stress condition using total RNA extracted from leaves samples (Described in Methods). Equal aliquots (20 μg) of total RNA were electrophoresed in each lane, transferred onto nylon membrane and probed with ^32^P-labeled gene specific DNA fragments (Left panels). Tubulin RNA probe was used as loading control on the same membranes. Desitometric analysis of the autoradiograms from three independent experiments (Right panels); *n *= 3 ± SE.

The expression level of the Cu/ZnSOD gene in *D. antarctica *plants exposed to stress conditions was examined by Northern analysis (Figure 4). Transcripts of Cu/ZnSOD were detected in all four conditions. The expression level of Cu/ZnSOD was 8 times higher in acclimated plants (4°C) than in non-acclimated plants (13°C) (Figure 4A), indicating that the expression of this gene is differentially regulated by temperature. This correlates to the finding that *D. antarctica *plants subjected to acclimation at 4°C showed an increase in the SOD enzymatic activity [27]. *D. antarctica *plants exposed to UV radiation have a 2 times higher transcript level than the non exposed plants (Figure 4B), this result suggest that this gene is involved in antioxidative defense in response to the UV stress.

## Conclusion

Given the absence of the sequence of signal peptide secretion in the cDNA sequence analysis of clone C13 isolated from the library of *D. antarctica*, we assume that the putative Cu/ZnSOD protein is probably located in the cytosol. The cDNA nucleotide sequence analysis shows that the *SOD *gene of *D. antarctica *encodes for a Cu/ZnSOD. In *D. antarctica*, the expression of Cu/ZnSOD is regulated by temperature and UV radiation, suggesting that this gene is involved in the antioxidant defense response under environmental stress. The Cu/ZnSOD may play an important role in the tolerance of *D. antarctica *to the harsh Antarctic environment, which has increased our interest to characterize the Cu/ZnSOD enzyme and to study its potential biotechnological application.

## Competing interests

The authors declare that they have no competing interests.

## Authors' contributions

JRSV: cloned the genomic sequence, performed experiments of molecular characterization and gene expression, bioinformatics analysis the nucleotide sequence and wrote the manuscript. JD: corrected the figures and reviewed the manuscript. AG: make the construction of cDNA library of *D. antarctica*. MG: participated in the coordination of the project. All authors read and approved the final manuscript.
